# Evaluation of changes in the tumor microenvironment after sorafenib therapy by sequential histology and ^18^F-fluoromisonidazole hypoxia imaging in renal cell carcinoma

**DOI:** 10.3892/ijo.2012.1624

**Published:** 2012-09-10

**Authors:** MASAHIRO MURAKAMI, SONGJI ZHAO, YAN ZHAO, NUSRAT FATEMA CHOWDHURY, WENWEN YU, KEN-ICHI NISHIJIMA, MITSUYOSHI TAKIGUCHI, NAGARA TAMAKI, YUJI KUGE

**Affiliations:** 1 Laboratory of Veterinary Internal Medicine, Graduate School of Veterinary Medicine, Hokkaido University, Kita-ku, Sapporo 060-0818;; 2 Departments of Nuclear Medicine and; 3 Tracer Kinetics and Bioanalysis, Graduate School of Medicine, Hokkaido University, Kita-ku, Sapporo 060-8638;; 4 Oral Diagnosis and Medicine, Department of Oral Pathobiological Science, Graduate School of Dental Medicine, Hokkaido University, Kita-ku, Sapporo 060-8586;; 5 Central Institute of Isotope Science, Hokkaido University, Kita-ku, Sapporo 060-0815, Japan

**Keywords:** tumor starvation, tumor hypoxia, anti-angiogenic therapy, ^18^F-fluoromisonidazole, positron emission tomography

## Abstract

The mechanistic dissociation of ‘tumor starvation’ versus ‘vascular normalization’ following anti-angiogenic therapy is a subject of intense controversy in the field of experimental research. In addition, accurately evaluating changes of the tumor microenvironment after anti-angiogenic therapy is important for optimizing treatment strategy. Sorafenib has considerable anti-angiogenic effects that lead to tumor starvation and induce tumor hypoxia in the highly vascularized renal cell carcinoma (RCC) xenografts. ^18^F-fluoromisonidazole (^18^F-FMISO) is a proven hypoxia imaging probe. Thus, to clarify early changes in the tumor microenvironment following anti-angiogenic therapy and whether ^18^F-FMISO imaging can detect those changes, we evaluated early changes in the tumor microenvironment after sorafenib treatment in an RCC xenograft by sequential histological analysis and ^18^F-FMISO autoradiography (ARG). A human RCC xenograft (A498) was established in nude mice, for histological studies and ARG, and further assigned to the control and sorafenib-treated groups (80 mg/kg, *per os*). Mice were sacrificed on Days 1, 2, 3 and 7 in the histological study, and on Days 3 and 7 in ARG after sorafenib treatment. Tumor volume was measured every day. ^18^F-FMISO and pimonidazole were injected intravenously 4 and 2 h before sacrifice, respectively. Tumor sections were stained with hematoxylin and eosin and immunohistochemically with pimonidazole and CD31. Intratumoral ^18^F-FMISO distribution was quantified in ARG. Tumor volume did not significantly change on Day 7 after sorafenib treatment. In the histological study, hypoxic fraction significantly increased on Day 2, mean vessel density significantly decreased on Day 1 and necrosis area significantly increased on Day 2 after sorafenib treatment. Intratumoral ^18^F-FMISO distribution significantly increased on Days 3 (10.2-fold, p<0.01) and 7 (4.1-fold, p<0.01) after sorafenib treatment. The sequential histological evaluation of the tumor microenvironment clarified tumor starvation in A498 xenografts treated with sorafenib. ^18^F-FMISO hypoxia imaging confirmed the tumor starvation. ^18^F-FMISO PET may contribute to determine an optimum treatment protocol after anti-angiogenic therapy.

## Introduction

Angiogenesis plays a critical role in tumor progression, invasion and metastasis (1,2). Thus, several anti-angiogenic agents are currently approved by the US Food and Drug Administration for use in cancer therapies that inhibit the VEGF pathways (3). Despite the fact that anti-angiogenic agent monotherapy has been proven effective for certain types of cancer (e.g., recurrent glioblastoma or ovarian cancer), it has shown only a modest target response rate in many cases (4,5). On the other hand, multiple clinical trials of metastatic colorectal cancer (6–8) and non-small cell lung cancer (9,10) have all confirmed that combination therapy with an anti-angiogenic agent and conventional chemotherapy or radiotherapy significantly improves outcomes in patients. The different responses to anti-angiogenic therapy have been clarified by two conflicting strategies using anti-angiogenic agents.

Conventionally, in anti-angiogenic therapy, inhibition of new vessel formation or destruction of existing vessels to reduce blood flow and starve the tumor (of its nutrients) to death is attempted (11). However, as evidence against this tumor starvation strategy, several preclinical studies have recently shown that direct or indirect blockade of VEGF signaling with pharmacological agents can transiently repair tumor vascular abnormalities, improve tumor oxygenation and blood flow and decrease interstitial fluid pressure (this process is referred to as vascular normalization strategy) (12,13). When an anti-angiogenic agent is able to starve tumor cells, it is considered effective for monotherapy. On the other hand, when an anti-angiogenic agent is able to normalize intratumoral blood flow, it is considered to have an enhancive effect in combination therapy. For example, vascular normalization increases the sensitivity to radiotherapy owing to the reoxygenation of tumor tissues (14), and improves drug delivery to target sites in chemotherapy owing to the increase in intratumoral blood flow. Thus, the mechanistic dissociation between tumor starvation and vascular normalization following anti-angiogenic therapy is a subject of intense debate in the field of cancer therapy and is the focus of much ongoing experimental research (15). The accurate evaluation of tumor responses after anti-angiogenic therapy is important for optimizing treatment strategy (16). However, the changes in the tumor microenvironment after anti-angiogenic therapy have yet to be clarified.

Recent advances in molecular imaging technologies have enabled us to non-invasively image tumor hypoxia using radiolabeled probes. Hypoxia imaging may reflect changes in the tumor microenvironment after anti-angiogenic therapy. However, only few studies by hypoxia imaging have been performed following anti-angiogenic therapy (16,17). Moreover, the associations between the findings of hypoxia imaging and changes in tumor microenvironment after anti-angiogenic therapy have yet to be clarified.

With the above background, the purpose of the present study was twofold: i) to clarify the changes in the tumor microenvironment at early time-points following anti-angiogenic therapy and ii) to determine whether ^18^F-fluoromisonidazole (^18^F-FMISO) hypoxia imaging can detect the changes in the tumor microenvironment after anti-angiogenic therapy.

Therefore, we evaluated the changes in the tumor microenvironment at early time-points after sorafenib treatment in RCC xenografts by sequential histological study and ^18^F-FMISO autoradiography (ARG).

## Materials and methods

### 

#### Cell lines and tumor xenograft models

**Figure 1.**
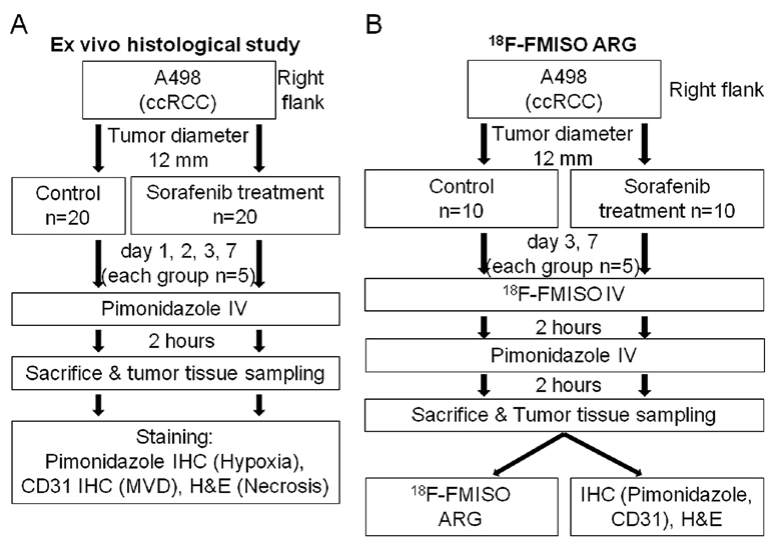
Experimental protocols of (A) *ex vivo* histological study and (B) ARG.

The human clear cell RCC (A498) cell line, which is the von Hippel-Lindau (VHL) mutant, was obtained from the European Collection of Cell Cultures (Salisbury, UK). Cells were maintained in RPMI-1640 medium (Invitrogen Life Technologies, Inc., Carlsbad, CA, USA) supplemented with 10% fetal bovine serum, penicillin-streptomycin and 0.03% glutamine and incubated in an atmosphere of 5% CO_2_ and 95% air at 37°C. All experimental protocols were approved by the Laboratory Animal Care and Use Committee of Hokkaido University. Nine-week-old male BALB/c athymic nude mice (Japan SLC, Inc., Hamamatsu, Japan) were used in all experiments. A human RCC xenograft model was established using the A498 cell line (1×10^7^ cells/0.l ml) by subcutaneous inoculation into the right flank of mice. When the tumors grew to 12 mm in diameter, the mice were randomly assigned to two groups (Fig. 1) for *ex vivo* histological study (n=40) and ARG study (n=20). Mice in the two groups were further assigned to the control and sorafenib treatment groups. Tumor growth curve was derived on Day 7 in the groups for *ex vivo* histological study. Tumor size was measured using a caliper every day prior to the treatment and tumor volume was calculated by the following formula: π/6 x larger diameter x (smaller diameter)^2^.

#### Treatment

Sorafenib (Nexavar; Bayer Pharmaceuticals Corp., West Haven, CT, USA), was used in all studies. Sorafenib (80 mg/kg) in a cremophor EL (Sigma, St. Louis, MO, USA)/ethanol (Pharmaco Products, Brookfield, CT, USA)/water (12.5:12.5:75) solution was administered daily by oral gavage. The cremophor EL/ethanol/water (12.5:12.5:75) solution was administered as the vehicle in the control groups.

#### Ex vivo histological study

The mice were randomly assigned to the control (n=20) and treatment groups (n=20) (Fig. 1): 4 time-points for each group (Days 1, 2, 3 and 7; n=5 for each time-point). The mice were anesthetized by diethyl ether inhalation and injected with pimonidazole hydrochloride [Hypoxyprobe™-1, 60 mg/kg; (Hypoxyprobe, Inc.; Burlington, MA, USA)] in the tail vein. Two hours later, these mice were sacrificed and the tumors were excised. The excised tumors were formalin-fixed and paraffin-embedded for subsequent histochemical staining.

#### Quantitative evaluation of histological staining

Formalin-fixed, paraffin-embedded, 4-*μ*m sections of the tumor were stained immunohistochemically with pimonidazole to assess hypoxia and with CD31 to assess microvessel density. Hematoxylin and eosin (H&E) staining was also performed to assess necrosis. To assess hypoxia, a Hypoxyprobe-1 Omni kit (Hypoxyprobe, Inc.) was used. Briefly, after deparaffinization and rehydration, the slides were initially immersed in a 10-mM citrate buffer solution and heated in boiled water for 20 min. After antigen retrieval, endogenous peroxidase activity was blocked for 10 min in methanol containing 0.3% hydrogen peroxide. Thereafter, sections were incubated with a rabbit polyclonal anti-pimonidazole antibody (PAb2627AP) diluted at 1:200 for 60 min. To assess microvessels, following deparaffinization and rehydration, the slides were initially immersed in a target retrieval solution (pH 9.0; Dako, Glostrup, Denmark) and heated in boiled water for 20 min. Following antigen retrieval, endogenous peroxidase activity was blocked for 10 min in methanol containing 0.3% hydrogen peroxide. Sections were then incubated with a rabbit polyclonal antibody to CD31 (ab28364) (Abcam; Cambridge, UK) diluted at 1:50 for 30 min. In immunohistochemical staining with both antibodies, the bound antibodies were visualized using the avidin/biotin conjugate immunoperoxidase procedure with a Histofine SAB-PO kit (Nichirei, Tokyo, Japan) and 3,3′-diaminobenzi-dine tetrahydrochloride. The slides were counterstained with Mayer’s hematoxylin solution (Wako, Osaka, Japan). To assess necrosis, H&E staining was performed. For the quantitative analysis of hypoxia, the percentage of the area positively stained by pimonidazole in the entire tumor cross section was calculated as the hypoxic fraction (% pimonidazole-positive area) using Image J. For the quantitative analysis of microvessel density, CD31-positive intratumoral microvessels were counted blindly under a microscope field (x400 objective magnification, 0.644 mm^2^ per field). More than 10 fields per section were randomly analyzed, excluding peripheral connective tissue and central necrotic tissue. Single CD31-positive endothelial cells without any visible lumen were not counted. The number of CD31-positive intratumoral microvessels was expressed as mean vessel density (MVD, vessels/field). Necrosis area was determined from H&E-stained consecutive sections using Image J.

#### ^18^F-FMISO autoradiography

In ARG, the mice were randomly assigned to the control group (n=10) and the treatment group (n=10): two time-points for each group (Days 3 and 7, n=5 for each time-point). The mice were anesthetized by diethyl ether inhalation and injected with 18.5 MBq of ^18^F-FMISO, which was synthesized as previously described (18,19). Two hours after ^18^F-FMISO injection, the mice were anesthetized by diethyl ether inhalation again and injected with pimonidazole (60 mg/kg) in the tail vein. Two hours after pimonidazole injection, these mice were sacrificed and the tumors were quickly excised. Each excised tumor was then sectioned to obtain two adjacent slices. One slice was embedded in Tissue-Tek medium (Sakura Finetechnical Co., Ltd., Tokyo, Japan) with the calf muscle a short distance away from the tumor slice and frozen in isopentane/dry ice for ARG. Formalin-fixed, paraffin-embedded specimens were prepared using the other slice for the subsequent quantitative staining analysis. For ARG, the frozen specimens were cut into 10-*μ*m sections with a CM3050-Cryostat (Leica Microsystems, Wetzlar, Germany) at 20°C. The tumor sections were placed in a phosphor image plate cassette, together with a set of calibrated standards (20) and an overnight ARG exposure was performed to detect the distribution of ^18^F-FMISO. The tumor sections (5 *μ*m) were stained with H&E for use as the reference to determine the regions of interest (ROIs) on the obtained autoradiograms. ARG images were analyzed using a computerized imaging analysis system (FLA 7000 Bio-Imaging Analyzer; Fuji Photo Film Co., Ltd., Tokyo, Japan). To quantitatively evaluate ^18^F-FMISO radioactivity, ROIs were placed to cover entire tumor tissues and muscles on each ARG image with reference to H&E-stained sections. The radioactivity in each ROI was calculated using the activity of the standards and converted to the percentage injected dose per kilogram body weight per square meter of tissue (% ID/m^2^ × kg body weight) (21,22).

#### Statistical analysis

All statistical analyses were carried out using StatView version 5.0 (SAS Institute, Inc.). All values are expressed as mean ± SD. One-factor repeated measures ANOVA was used to assess the significance of differences in trends of tumor volume between the control and treatment groups. In the evaluation of staining and ^18^F-FMISO ARG, the Mann-Whitney U test was used to assess the significance of differences between the control and treatment groups at each time-point. A p<0.05 was considered to indicate statistically significant differences.

## Results

### Ex vivo histological study

#### Tumor volume change

**Figure 2.**
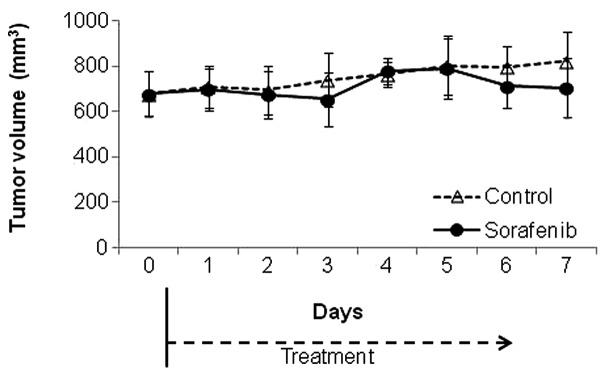
Tumor growth curve. Dotted arrow, treatment period.

The tumor growth curve is shown in Fig. 2. There was no significant difference in tumor volume between the control and sorafenib-treated groups during the study period until Day 7 (p=0.24).

#### Quantitative evaluation of histological staining

**Figure 3.**
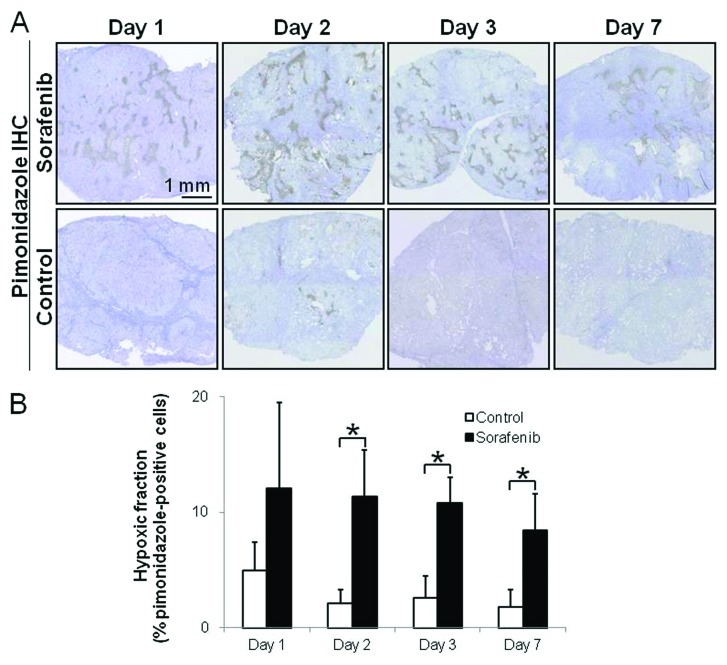
(A) Microscopy images and (B) quantitative analysis of hypoxia on Days 1, 2, 3 and 7 after treatment with vehicle or sorafenib. (A) Representative images of pimonidazole staining (brown staining in pimonidazole-positive cells). (B) Mean hypoxic fraction ± SD. ^*^p<0.01 vs. control group at the same time-point.

Fig. 3 shows representative images and the quantitative evaluation of hypoxia in tumor tissues on Days 1, 2, 3 and 7 after the treatment with the vehicle or sorafenib. In the sorafenib-treated groups, apparent pimonidazole-positive hypoxic areas were visually observed at various time-points (Fig. 3A). In the control group, even in tumors that were sufficiently large, only few hypoxic regions were detectable at any time-point (Fig. 3A). The hypoxic fractions (% pimonidazole-positive cells) were significantly increased by 5.4-, 4.2-and 4.7-fold on Days 2, 3 and 7 after treatment with sorafenib, respectively, compared with the control group (Fig. 3A). The hypoxic fractions in tumors were 4.9±2.5 and 12.0±7.5 (%) on Day 1 (p= 0.17), 2.1±1.2 and 11.3±4.0 (%) on Day 2 (p<0.01), 2.6±1.9 and 10.8±2.3 (%) on Day 3 (p<0.01) and 1.8±1.5 and 8.4±3.2 (%) on Day 7 (p<0.05) in the control and sorafenib-treated groups, respectively (Fig. 3B).

**Figure 4.**
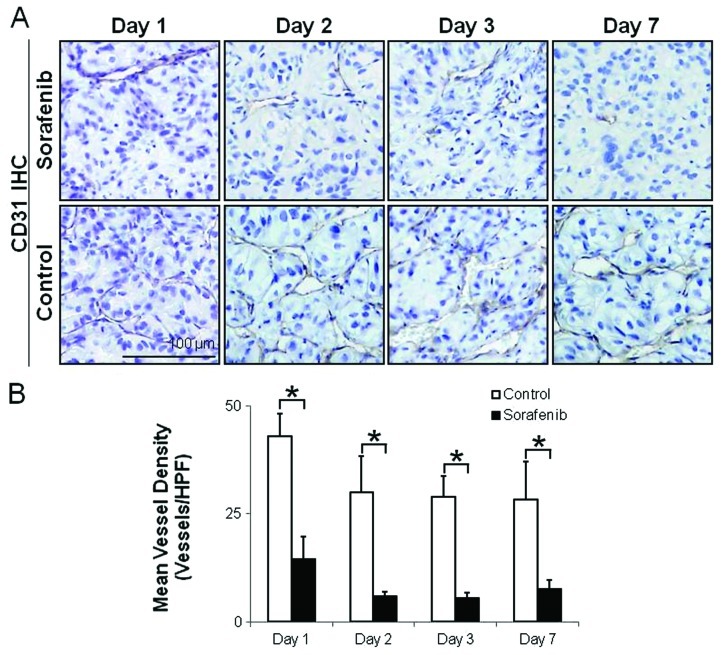
(A) Microscopy images and (B) quantitative analysis of intratumoral microvessels on Days 1, 2, 3 and 7 after treatment with vehicle or sorafenib. (A) Representative images of CD31 staining (brown staining in CD31-positive cells). (B) Mean vessel densities (MVDs) ± SD. ^*^p<0.01 vs. control group at the same time-point.

Fig. 4 shows representative images and the quantitative evaluation of microvessels. The number of CD31-positive intratumoral microvessels was markedly high in the control group (Fig. 4A). The MVDs significantly decreased by 66, 80, 81 and 73% on Days 1, 2, 3 and 7 after treatment with sorafenib, respectively, compared with the control group (Fig. 4A). The MVDs in tumors were 43.0±5.3 (vessels/HPF) and 14.5±5.4 (vessels/HPF) on Day 1 (p<0.01), 30.0±8.3 (vessels/HPF) and 5.9±1.0 (vessels/HPF) on Day 2 (p<0.01), 29.0±4.8 (vessels/HPF) and 5.5±1.2 (vessels/HPF) on Day 3 (p<0.01) and 28.2±8.9 (vessels/HPF) and 7.5±2.1 (vessels/HPF) on Day 7 (p<0.05) in the control and sorafenib-treated groups, respectively (Fig. 4B).

**Figure 5.**
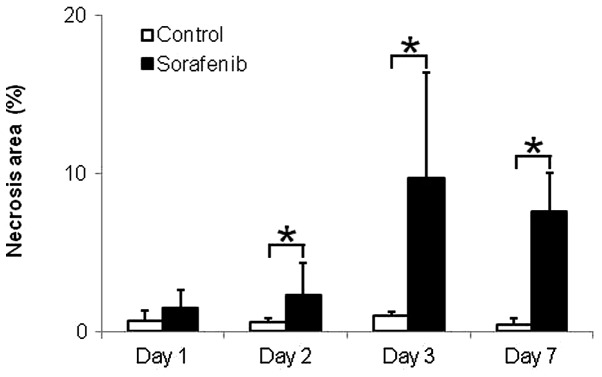
Quantitative analysis of necrosis on Days 1, 2, 3 and 7 after treatment with vehicle or sorafenib. Necrosis areas (%) ± SD. ^*^p<0.01 vs. control group at the same time-point.

Fig. 5 shows the quantitative evaluation of necrosis in tumor tissues. The necrosis areas were significantly increased by 3.8-, 9.7- and 15.2-fold on Days 2, 3 and 7 after treatment with sorafenib, respectively, compared with those in the control group (Fig. 5). The necrosis areas in tumors were 0.7±0.6 (%) and 1.5±1.2 (%) on Day 1 (p<0.60), 0.6±0.2 (%) and 2.3±2.0 (%) on Day 2 (p<0.05), 1.0±0.3 (%) and 9.7±6.7 (%) on Day 3 (p<0.01) and 0.5±0.4 (%) and 7.6±2.5 (%) on Day 7 (p<0.05) in the control and sorafenib-treated groups, respectively (Fig. 5).

#### ^18^F-FMISO ARG

**Figure 6.**
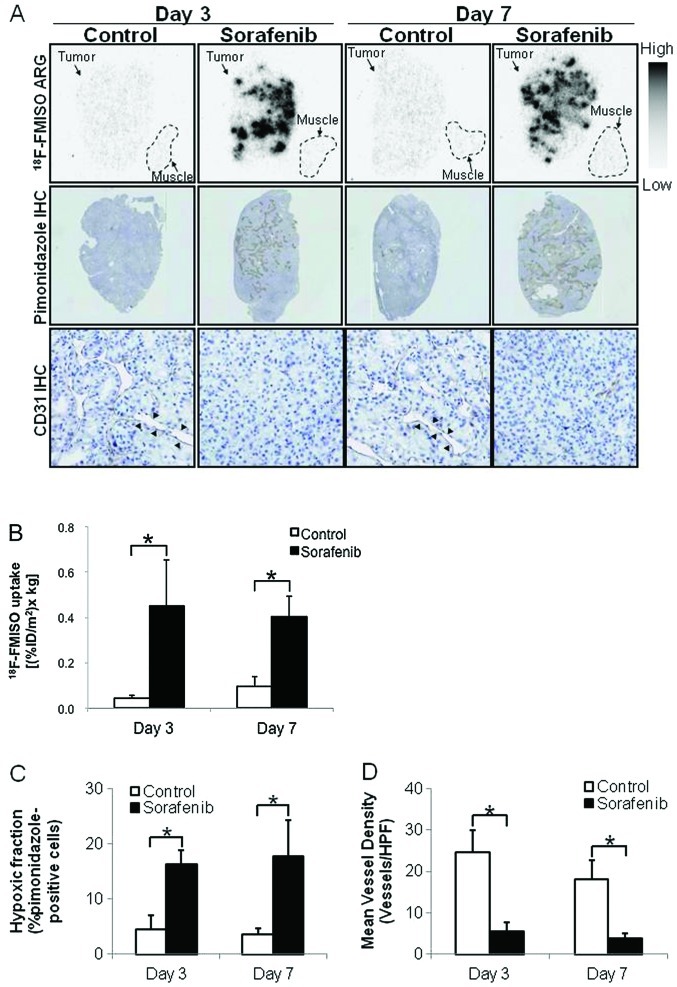
(A) Representative images and (B–D) quantitative analysis of (A and B) intratumoral ^18^F-FMISO and immunohistochemical stainings of (A and C) pimonidazole and (A and D) CD31 on Days 3 and 7 after treatment with vehicle or sorafenib. (A) Comparison among ^18^F-FMISO ARG, pimonidazole IHC and CD31 IHC images. Representative ^18^F-FMISO ARG images, pimonidazole IHC (brown staining in pimonidazole-positive cells) and CD31 IHC (black arrowhead around microvessels, staining in CD31-positive cells). Dotted line shows muscle. (B) Mean ^18^F-FMISO accumulation levels ± SD. (C) Mean hypoxic fraction ± SD. (D) Mean vessel densities (MVDs) ± SD. ^*^p<0.01 vs. control group at the same time-point (B–D).

Considering the results of the *ex vivo* histological studies, ^18^F-FMISO ARG was performed on Days 3 and 7 after the treatment. Fig. 6 shows representative images and quantitative evaluation by ^18^F-FMISO ARG. Hypoxia and microvessel density were also evaluated using sections adjacent to those used in ARG (Fig. 6C and D). ^18^F-FMISO ARG showed visually the increase in tumor hypoxic area, which is similar to the finding of pimonidazole IHC (Fig. 6A). CD31 IHC showed a decrease in tumor microvessel density after treatment with sorafenib (Fig. 6A). In the control groups, ^18^F-FMISO ARG showed a low extent of intratumoral ^18^F-FMISO distribution, which was similar to that of muscle (Fig. 6A). The intratumoral ^18^F-FMISO distribution extent significantly increased by 10.2- and 4.1-fold on Days 3 and 7 after treatment with sorafenib, respectively, compared with the control group (Fig. 6B). The levels of ^18^F-FMISO accumulation in tumors were 0.044±0.013 and 0.451±0.201 (% ID/m^2^ x kg body weight) on Day 3 (p<0.01) and 0.097±0.040 and 0.400±0.094 (% ID/m^2^ x kg body weight) on Day 7 (p<0.01) in the control and sorafenib-treated groups, respectively (Fig 6B). The hypoxic fractions (% pimonidazole-positive cells) in tumor tissues significantly increased by 3.5- and 4.8-fold on Days 3 and 7 after treatment with sorafenib, respectively, compared with the control group (Fig. 6C). The hypoxic fractions in tumors were 4.6±2.4 and 17.8±6.5 (%) on Day 3 (p<0.01) and 4.6±2.4 and 16.2±2.5 (%) on Day 7 (p<0.01) in the control and sorafenib-treated groups, respectively (Fig. 6C). The MVDs in tumor tissues significantly decreased by 77 and 78% on Days 3 and 7 after treatment with sorafenib, respectively, compared with the control groups (Fig. 6D). The MVDs in tumor tissues were 24.7±5.2 and 5.6±2.2 (vessels/HPF) on Day 3 (p<0.01) and 18.0±4.7 and 3.9±1.2 (vessels/HPF) on Day 7 (p<0.01) in the control and sorafenib-treated groups, respectively (Fig. 6D).

## Discussion

There are two major findings of this study. First, we showed that the tumor hypoxic fraction increased significantly after only 2 days of sorafenib treatment, following the markedly reduced tumor microvessel density after only 1 day of sorafenib treatment in A498 xenografts (Figs. 3 and 4). Subsequently, the necrosis area increased after 2–3 days of sorafenib treatment in A498 xenografts (Fig. 5), although tumor volume did not change after sorafenib treatment, at least after 7 days of sorafenib treatment (Fig. 2). This finding indicates that tumor starvation by anti-angiogenic therapy occurs following sorafenib treatment in A498 xenografts and it is detected as tumor hypoxia. Second, we showed that ^18^F-FMISO ARG detects the changes in the tumor microenvironment as shown by a significant enhancement of tumor hypoxia after 3 and 7 days of sorafenib treatment in A498 xenografts (Fig. 6). This finding indicates that ^18^F-FMISO can detect the tumor starvation process after anti-angiogenic therapy from a rapid enhancement of tumor hypoxia. Therefore, findings of this study suggest that ^18^F-FMISO PET is a promising tool for non-invasive evaluation of tumor starvation after anti-angiogenic sorafenib treatment in highly vascularized RCC tumor xenografts.

Conventionally, the therapeutic responses of tumor have been assessed by serial tumor size measurements, most notably using the Response Evaluation Criteria in Solid Tumors guidelines (23). However, preclinical assessment of anti-angiogenic agents has highlighted limitations associated with standard morphologic measurements. Thus, tumor responses may be better assessed by functional measurements, which may be more appropriate than size measurements (17,24,25).

In the evaluation of functional measurements, which reflect tumor responses after anti-angiogenic therapy, two conflicting hypotheses about changes in the tumor microenvironment following anti-angiogenic therapy raise a problem. First is the conventional tumor starvation strategy, by which inhibition of new vessel formation or destruction of existing vessels to starve the tumor from its nutrients and oxygen delivery is attempted (11). Previous studies also showed an enhancement of tumor hypoxia after treatment with anti-angiogenic agents including sorafenib (16,17,26). The other is a new vascular normalization theory, which states that pruning hyperpermeable immature tumor vessels can transiently repair these vascular abnormalities and improve tumor oxygenation after treatment with several anti-angiogenic agents including bevacizumab and sunitinib (12,27). Although the mechanisms underlying these conflicting processes in the tumor microenvironment following anti-angiogenic therapy remain unclear, changes in the tumor oxygen status and/or vascular function may distinguish these conflicting processes.

In the present study, changes in the hypoxia status examined by pimonidazole immunohistochemical analysis occurred at a very early time-point, i.e., after only 2 days of sorafenib treatment *ex vivo* (Fig. 3). The number of tumor microvessels significantly decreased after only 1 day of sorafenib treatment (Fig. 4). As a result, the necrosis area significantly increased after 2 days of sorafenib treatment (p<0.05) and the increase was more obvious after 3 days of sorafenib treatment (p<0.01) (Fig. 5). Since necrosis development following an acute decrease in the count of microvessels after anti-angiogenic therapy indicates tumor starvation (28–31), the sequential changes in the tumor microenvironment observed in the present study indicate the existence of tumor starvation and tumor hypoxia may reflect the tumor starvation status. Our findings are in line with those of Chang *et al*, who showed the enhancement of tumor hypoxia and decrease in the number of microvessels after 3 days of sorafenib treatment in VHL mutant 786-O ccRCC xenografts (26). However, our study is the first to show the sequential changes in the tumor microenvironments, which start from the decrease in the number of microvessels to induce tumor hypoxia and necrosis at early time-points after sorafenib treatment in highly vascularized RCC xenografts. Thus, the present sequential histological analyses of microvessels, hypoxia and necrosis may clarify the tumor starvation process following anti-angiogenic therapy.

Two experimental materials appear to contribute to the rapid increase in tumor hypoxia. The first is the A498 cell line, which is a VHL tumor suppressor gene mutant and in which hypoxia-inducible factor (HIF)-2α is activated, although HIF-1α is absent (32). A hallmark of RCC is the frequent loss of VHL, which is a key regulator of HIF (33–35) and VEGF production (36). Loss of VHL results in the upregulation of VEGF production and induction of tumor angiogenesis (37). Thus, RCC with loss of VHL is a highly vascularized and treatment-resistant tumor (38) and is one of the most studied tumors treated with anti-angiogenic therapy (39,40). Since A498 is highly vascularized, anti-angiogenic therapy tends to induce acute tumor hypoxia. The other material is sorafenib, which is a small-molecule multikinase inhibitor that has been approved for treating advanced RCC (41). As sorafenib has a strong anti-angiogenic effect with suppression of the vascular endothelial growth factor receptor (VEGFR) and platelet-derived growth factor receptor (PDGFR), it is markedly effective for RCC (26,42). It is indicated that this strong anti-angiogenic agent induces acute tumor hypoxia in this highly vascularized tumor. The results of the present study show that the rapid enhancement of tumor hypoxia almost exclusively reflects tumor starvation status and tumor hypoxia evaluation has great possibility in that it reflects direct anti-angiogenic treatment effect and is useful for determining patient-specific treatment protocols.

To identify predictive biomarkers for anti-angiogenic therapy, various imaging techniques are being examined, including perfusion imaging in dynamic contrast-enhanced magnetic resonance imaging (DCE-MRI) (43,44). Several clinical trials have shown a decrease in tumor perfusion in response to anti-angiogenic treatment (45). Perfusion imaging is suitable for evaluating the vascular status. However, DCE-MRI does not directly assess the cellular statuses including tumor hypoxia and necrosis. Hypoxia imaging may directly evaluate tumor cell status and may distinguish tumor hypoxic areas from necrotic areas. Thus, both perfusion and hypoxia imaging techniques should promote our understanding of the tumor microenvironment following anti-angiogenic therapy and an optimum imaging modality should be chosen according to the purpose and situation.

There are several methods that indicate the tumor oxygen status. One of the most reliable methods of evaluation of hypoxia in tumor is pimonidazole immunohistochemical analysis (46). However, pimonidazole immunohistochemical analysis is an invasive and two-dimensional qualitative method. ^18^F-FMISO is the most widely used PET probe for imaging tumor hypoxia. Both the hypoxia probes ^18^F-FMISO and pimonidazole are imidazole derivatives. The 2-nitroimidazole moiety in these compounds is considered to be reduced by nitroreductase enzymes in a hypoxic environment and trapped in hypoxic tumor cells; therefore, ^18^F-FMISO and pimonidazole accumulate in similar regions in tumors (47,48). Thus, ^18^F-FMISO PET can quantitatively evaluate three-dimensional hypoxia regions (49,50). As mentioned above, the present findings on the intratumoral ^18^F-FMISO distribution indicate the significant enhancement of tumor hypoxia after sorafenib treatment in A498 xenografts, which is consistent with the findings of the concurrent histological and *ex vivo* histological analyses (Fig. 6). Thus, ^18^F-FMISO PET findings may reflect tumor starvation after anti-angiogenic therapy *in vivo* at an early time-point.

In conclusion, the sequential histological changes of the tumor microenvironment clarified tumor starvation in A498 tumor xenografts treated with sorafenib and ^18^F-FMISO hypoxia imaging confirmed this tumor starvation. Unlike vascular normalization, which shows an attenuation of tumor hypoxia, the rapid enhancement of tumor hypoxia directly reflects the effect of treatment with sorafenib alone. Thus, the assessment of tumor hypoxia following anti-angiogenic therapy is important for selecting optimum treatment protocols, i.e., between anti-angiogenic therapy alone and combination therapy. ^18^F-FMISO PET may be used to assess tumor hypoxia following anti-angiogenic therapy and may contribute to determining optimum treatment protocols for cancer patients on anti-angiogenic therapy.
